# Mucin 5B in saliva and serum of patients with oral lichen planus

**DOI:** 10.1038/s41598-017-12157-1

**Published:** 2017-09-21

**Authors:** Farzaneh Agha-Hosseini, Mojtaba Imanpour, Iraj Mirzaii-Dizgah, Mahdieh-Sadat Moosavi

**Affiliations:** 1 Dental Research Center, Dentistry Research Institute, Department of Oral and Maxillofacial Medicine, Tehran University of Medical Sciences, Tehran, Iran; 2 Faculty of Dentistry, Tehran University of Medical Sciences, Tehran, Iran; 3 Department of Physiology, Faculty of Medicine, Aja University of Medical Sciences, Tehran, Iran

## Abstract

Oral lichen planus (OLP) is among the most common oral diseases. Its etiopathogenesis has yet to be clearly identified. OLP patients complain of mouth dryness. This study aimed to assess the level of Mucin 5B in OLP patients with xerostomia. This study was conducted on 30 OLP patients and 30 healthy individuals. In addition to patient complaint of mouth dryness, xerostomia was assessed by tongue blade and lipstick tests. Stimulated and unstimulated saliva were collected in plastic vials by spitting method. Level of Mucin 5B was measured by ELISA. Unstimulated saliva flow was significantly lower in OLP patients (P = 0.0001). Stimulated saliva flow was not significantly different between the two groups (P > 0.05). Level of Mucin 5B in unstimulated saliva was significantly lower in OLP group (P = 0.0001) while it was not significantly different in stimulated saliva of the two groups (P > 0.05). Level of Mucin 5B was significantly higher in serum of OLP patients (P = 0.016). Both saliva flow and level of Mucin 5B decrease in OLP patients. Since Mucin 5B is effective for wetting and lubrication of the oral cavity, this result can suggest a possible reason for mouth dryness in OLP patients.

## Introduction

Patients with oral lichen planus (OLP) comprise a large fraction of patients presenting to oral medicine departments. Lichen planus (LP) is a chronic autoimmune cell-mediated inflammatory disease, affecting the skin and oral mucosa. It is characterized by some reactions in the basal epithelial layer, which are associated with hypersensitivity reaction type IV, often involving T lymphocytes^1^. The middle-aged and elderly are more commonly affected, and OLP is more common in females^2^. It is among the most common oral mucosal diseases^1^ and manifests in different keratotic forms (plaque, reticular). It may also involve non-keratotic areas (atrophic, erythematous, erosive and bullous types) in the oral cavity, and can eventually form an ulcer. In keratotic OLP, patients complain of roughness and irregularity of lesions, while in non-keratotic, patients complain of pain and burning sensation, which depends on the extent and depth of lesions as well as the patient’s tolerance. The severity of pain may vary from mild to severe, and it may even interfere with speaking, eating and deglutition of patients^3^. Despite several studies on the etiopathogenesis of OLP, this has yet to be clearly understood. Because of this, its treatment is mainly symptomatic^4^. Moreover, OLP has premalignant potential and its exact mechanism of action has yet to be fully understood. Some OLP patients complain of xerostomia^5^; however, the reason is still unclear. Consequently, researchers have focused on mouth dryness in OLP patients, although studies on this topic are limited, compared to those on etiopathogenesis or treatment of OLP. Non-keratotic (atrophic, erosive) forms associated with mouth dryness result in greater patient complaints^6,7^.

Saliva is an exocrine secretion comprising 99% water and 1% organic and inorganic compounds. Mucin is one constituent of the saliva. It is a high molecular weight, glycosylated protein produced by epithelial tissue, and secreted by mucosal surfaces. It contains 18% protein, 72% carbohydrates, 1.4% sulfate and 1.45 phosphate. A key property of mucin is its ability to form gel^8^. It plays a prominent role in oral health. It serves as a lubricant, and protects oral mucosa. It helps mastication, deglutition and speech. It also has antimicrobial properties, and serves as the first defense barrier in epithelial tissue. It is part of the non-immune defense system. and bonds to pathogens^9^. The amount and role of mucin associated with different diseases, such as xerostomia in patients with autoimmune conditions such as Sjogren’s syndrome and Graves’ disease, have been evaluated^10,11^. Since Sjogren’s syndrome is currently the most important autoimmune disease causing xerostomia^12^, studies on etiopathogenesis of mouth dryness in this syndrome can also be evaluated for OLP (which is also an autoimmune disease that is more common in females). Since the exact etiology of OLP is not clear, finding etiology of its manifestation such as dry mouth may provide insight into OLP pathogenesis. Also the presence of sufficient saliva is essential to proper oral hygiene and wound healing in these patients^13^. There are two major salivary mucins, the high mulocular mass (MG1) and the low molecular mass (mg2) mucins. The MG1 mucin core is encoded by mucin5B gene^14^. Mucin5B is the predominant mucin in the salivary gland and its alteration in other autoimmune diseases such as Sjogren syndrome has been reported in previous studies^10^. So, as a first step in assessing salivary mucin changes, this study aimed to assess the flow of stimulated and unstimulated saliva and level of Mucin 5B in stimulated and unstimulated saliva and serum of patients with OLP. The larger salivary mucin MUC5B is related to the perception of xerostomia due to being held within the moisture in the mucosa^15^.

## Materials and Methods

This study was approved by the Ethics Committee of our university (code: IR.TUMS.VCR.REC.1395.299) and all methods were performed in accordance with the relevant guidelines. Patients in both test and control groups signed informed consent forms prior to participation in the study.

The study was conducted on 30 patients with OLP, and 30 healthy controls, presenting to the Oral Medicine Department of our Dental School. Patients with OLP included 11 males and 19 females in the age range of 30–82 years (mean age of 50 years), who were evaluated as our test group. Per each patient in the test group, one age- and sex-matched (frequency matched) healthy control was included. The control group also contained 11 males and 19 females in the age range of 25–55 years (mean age of 41 years).

The inclusion criteria were: confirmed diagnosis of OLP according to the World Health Organization (WHO) criteria in 2003; mouth dryness complaint; and positive tongue blade and lipstick tests^13^. Patients with OLP underwent thyroid hormone, zinc, vitamin B12, folic acid and CBC (diff) tests.

Definite diagnosis of OLP was made based on the criteria set by WHO, clinical examinations (bilateral presence of lesions, presence of papular or reticular patterns in parts of lesion), and confirmation of disease by biopsy [hydropic degeneration of basal layer cells and band-like presence of mononuclear infiltrates (lymphocytes) in superficial areas of the connective tissue]^13^.

Healthy controls had no history or family history of xerostomia, and their clinical examination was unremarkable. They had no intraoral lesions and were negative for tongue blade and lipstick tests.

The exclusion criteria (for both tests and controls) were: immunologic diseases, diabetes mellitus, malignancies, history of radiotherapy in the head and neck region, smoking, diseases that decrease saliva flow (such as Sjogren’s syndrome), use of anti-Parkinson’s medications, use of immunosuppressants or immunomodulators, use of medications causing xerostomia (antihistamines, anticholinergics, anticonvulsants, muscle relaxants, tranquilizers, sleeping pills, cytotoxic drugs and three-cyclic anti-depressants), patients with lesions next to amalgam fillings, lichenoid drug reactions, pregnancy or nursing, inflammatory diseases, or oral fungal diseases. Patients with evidence of dysplasia in their pathology report were also excluded.

A questionnaire (Tables 1 and 2)^16,17^ was used to assess mouth dryness and its severity in the two groups. Patients not eliminated by the exclusion criteria, and who gave a minimum of three positive answers to questions in Table 1, were included in the case group. Subjects with no positive responses were recruited as controls. (A minimum of three positive responses was required because questions 2 and 9 are not specific to xerostomia, and may be seen in some other conditions^18^).

**Table 1:** Questionnaire used for selection of subjects with xerostomia.

1. Does your mouth feel dry when eating a meal?
2. Do you have difficulties swallowing any foods?
3. Do you need to sip liquids to aid in swallowing dry foods?
4. Does the amount of saliva in your mouth seem to be reduced most of the time?
5. Does your mouth feel dry at night or on waking?
6. Does your mouth feel dry during the daytime?
7. Do you chew gum or use candy to relieve oral dryness?
8. Do you usually wake up thirsty at night?
9. Do you have problems in tasting food?
10. Does your tongue burn?

Response options: yes/no.

**Table 2:** The Xerostomia Inventory (XI).

I sip liquids to help swallow food.
My mouth feels dry when eating a meal.
I get up at night to drink.
My mouth feels dry.
I have difficulty in eating dry foods.
I suck sweets or cough lollies to relieve dry mouth.
I have difficulties swallowing certain foods.
The skin of my face feels dry.
My eyes feel dry.
My lips feel dry.
The inside of my nose feels dry.

Response options: never (score of 1), hardly (2), occasionally (3), fairly often (4), very often (5).

To prevent the effect of circadian rhythm on the saliva, saliva samples were collected between 10 a.m. and 12 p.m. To collect unstimulated saliva samples, the patients were asked to refrain from eating and drinking for 60 to 90 minutes prior to sampling. Saliva was collected when the patients were at rest by spitting into plastic vials. The patients were asked to first swallow their saliva and then tip their head down, collect the saliva in their mouth and then spit it into a sterile graded vial.

To collect stimulated saliva, patients were requested to chew equal pieces of mastic gum for one minute and then throw it out, swallow their saliva and spit into a vial. Next, 5cc of blood were also drawn from each patient. Saliva collection in patients was done after confirming their diagnosis, and prior to initiation of treatment.

The samples were then centrifuged (2500 g, 10 min), and saliva (without sputum) and serum were transferred to separate microtubes. The samples were frozen at −70 °C. After completion of sampling, the samples were sent to a lab to determine the level of Mucin 5B by ELISA using a kit (EASTBIOPHARM, Hangzhou, China).

### Data availability statement

The datasets generated during the current study are available from the corresponding author on reasonable request.

## Results

After applying the inclusion and exclusion criteria, 30 OLP patients and 30 healthy controls were evaluated. Statistical analysis of the results showed that unstimulated saliva flow was significantly lower in OLP patients (P = 0.008). Stimulated saliva flow in the OLP group was significantly lower than that in controls (P = 0.035).

Unstimulated saliva flow and age correlated significantly – as age of patient increased, unstimulated saliva flow decreased (P = 0.008). Stimulated saliva flow showed an equivalent correlation (P = 0.002).

Level of Mucin 5B in unstimulated saliva was significantly lower in OLP group (P = 0.0001). The level of Mucin 5B in stimulated saliva of OLP patients was also lower than that in controls, but the difference was not statistically significant (P = 0.944).

The level of Mucin 5B in unstimulated saliva had no significant association with gender (P = 0.08). The association of Mucin 5B in stimulated saliva and gender was also not significant (P = 0.357). The level of Mucin 5B in unstimulated saliva and age were not significantly correlated (P = 0.894). The association of level of Mucin 5B in stimulated saliva and age was not significant either (P = 0.473).

**Figure 1:**
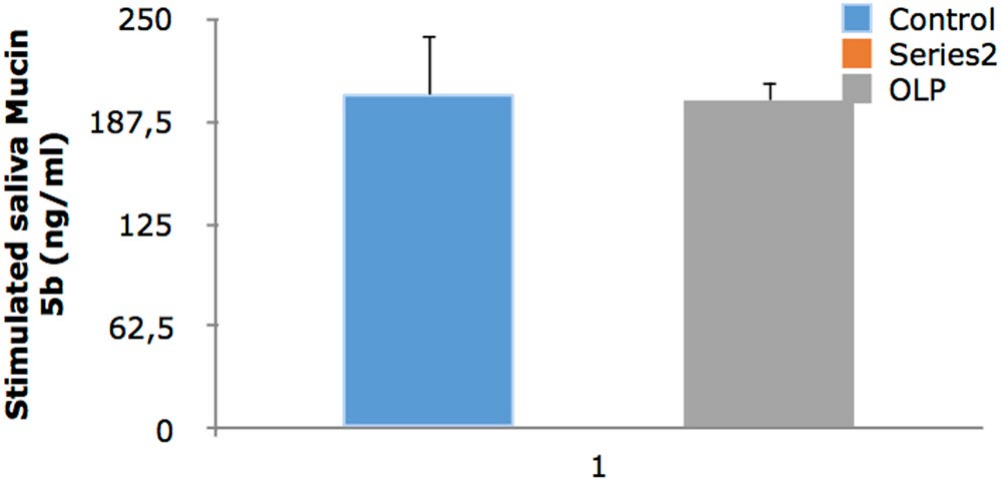
Level of Mucin 5B in stimulated saliva.

**Figure 2:**
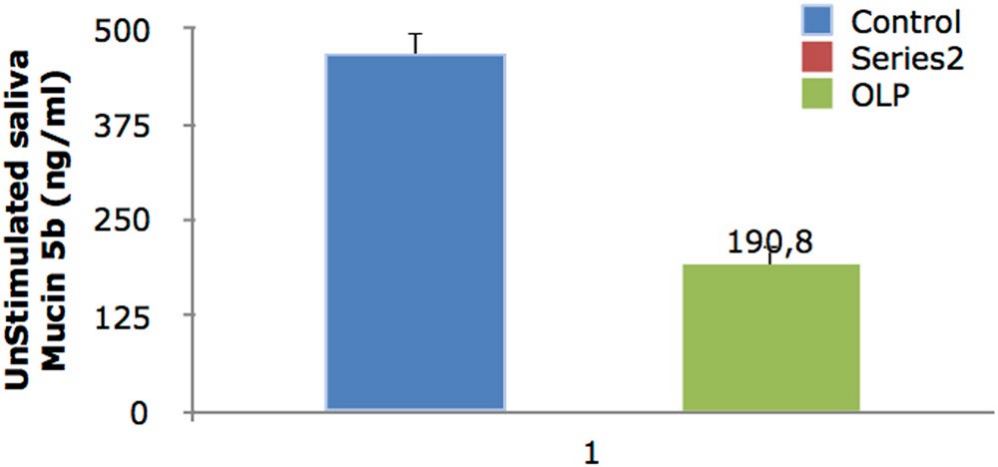
Level of Mucin 5B in unstimulated saliva.

**Figure 3:**
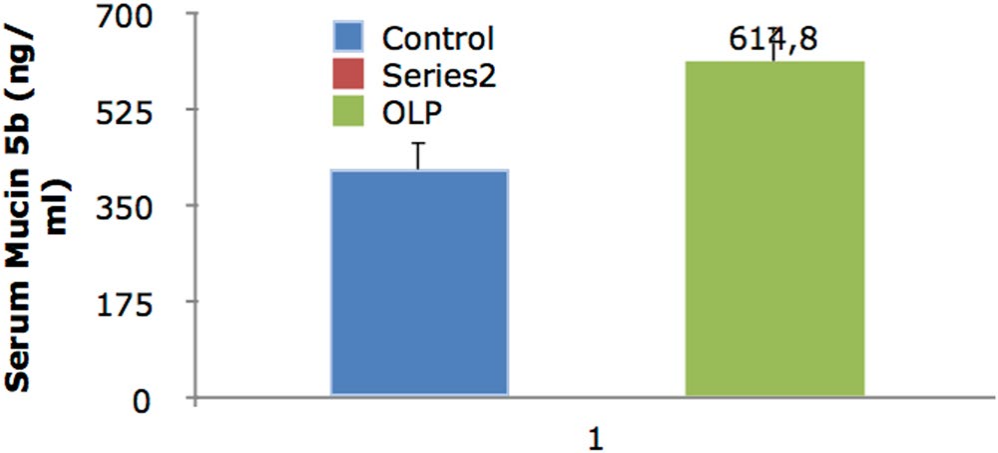
Level of Mucin 5B in serum. *Indicates statistically significant differences.

The level of Mucin 5B in the serum of OLP patients was significantly higher than that in controls (P = 0.016). The frequency of mouth dryness (based on patients questionnaire responses) was significantly higher in OLP patients (P = 0.0001). The release of Mucin 5B (amount of Mucin 5B multiplied by the saliva flow) was significantly lower in the stimulated saliva of OLP patients (P = 0.044). The release of Mucin 5B in unstimulated saliva was significantly lower in OLP patients (P = 0.001). A significant inverse correlation existed between the severity of xerostomia and level of Mucin 5B in unstimulated saliva (P = 0.0001, r = 0.513). No significant association was noted between the severity of xerostomia and the level of Mucin 5B in stimulated saliva (P = 0.156, r = 0.185). The severity of xerostomia was weakly correlated with the level of Mucin 5B in serum (P = 0.018, r = 0.307). Level of Mucin 5B in serum and saliva is shown in Table 3 and Figs 1–3.

**Table 3:** Level of Mucin 5B in serum and stimulated and unstimulated saliva of OLP patients and controls.

	Control	Case	P-value
Mucin 5B in serum (ng/ml)	417.1 ± 49.2	614.8 ± 63.0	0.016*
Mucin 5B in stimulated saliva (ng/ml)	203.1 ± 37.3	200.4 ± 11.5	0.944
Mucin 5B in unstimulated saliva (ng/ml)	466.1 ± 28.1	190.8 ± 26.0	0.0001*
Released Mucin 5B in unstimulated saliva (ng/min)	334.7 ± 42.6	79.6 ± 10.6	0.0001*
Released Mucin 5B in stimulated saliva (ng/min)	274.2 ± 72.5	122.1 ± 14.3	0.044*
Unstimulated saliva flow (ml/min)	0.72 ± 0.08	0.45 ± 0.06	0.008*
Stimulated saliva flow (ml/min)	1.27 ± 0.26	0.68 ± 0.07	0.035*

Data were reported as mean ± standard deviation.

*Indicates significant differences. P < 0.05 was considered statistically significant.

## Discussion

This study was conducted on 30 OLP patients and 30 matched healthy controls, to determine the level of Mucin 5B in serum and saliva as well as the saliva flow.

OLP is a chronic autoimmune T-lymphocyte dependent inflammatory disease with a prevalence of 0.1–4%; 0.5–3% of these lesions show dysplastic changes. WHO considers OLP as a lesion with premalignant potential. Due to its unknown etiology, its treatment is challenging, and the main focus is on elimination of mucosal lesions and alleviating patient symptoms such as pain and xerostomia^19,20^.

Oral hygiene affects lichen planus. So oral hygiene and saliva play important roles in the status of patients with autoimmune diseases such as OLP^21^. Saliva has antibacterial, antiviral and antifungal activity; it has a lubricating and wetting effect, and protects the mucosa; it plays a role in soft tissue healing. It is necessary for dental health and pH balance. Impaired saliva flow and change in its composition increase the susceptibility to caries, periodontal disease and oral lesions^22^.

Our study showed that unstimulated saliva flow was significantly lower in the case compared to the control group. Stimulated saliva flow was also lower in patients with OLP, but not significantly. This finding was in line with the results of previous studies regarding reduction in saliva flow in patients with OLP. The reason for such a reduction in saliva flow has yet to be fully elucidated. A previous study on cholinergic muscarinic receptors (MR3) in salivary glands of OLP patients showed a significant reduction in number of these receptors (affecting saliva output) in salivary glands of patients with OLP^13^.

Mucin present in saliva has a lubricating, wetting and softening effect. It protects the oral tissues and enhances mastication, deglutition and speaking ability. It also has an antibacterial effect^9^. Mucin 5B is a large-molecule glycoprotein and is the most abundant type of Mucin in the saliva and oral cavity^23^. The mucin5B isolated from whole saliva and the different salivary glands are varied in size which is because of a variable number of subunits in the one piece oligomeric mucins^24^. This mucin has different glycosylated low- and high-charge glycoforms. In the oral cavity, the high charged forms are secreted from the palatal glands, while the lower charged derived from the sublingual and submandibular glands^25^.

Alliende *et al*. measured the level of a Mucin 5B derivative in the saliva of patients with Sjogren’s syndrome, and found that its level was lower than normal in these patients. They concluded that in addition to decreased saliva flow, change in its composition and reduction in level of Mucin 5B can also be responsible for mouth dryness in patients with Sjogren’s syndrome^10^.

In 2012, Dijekma *et al*. evaluated 29 patients undergoing head and neck radiotherapy and found that the level of Mucin 5B was higher in patients with no xerostomia, or those with slight mouth dryness. They concluded that reduction in Mucin 5B may be a possible reason for xerostomia^15^. They also called for further studies on association of Mucin 5B and xerostomia.

To the best of the authors’ knowledge, no previous study has assessed the association of Mucin 5B and xerostomia on patients with OLP. Change in saliva composition may be responsible for xerostomia. Pathogenesis and etiology of this disease may be the reason for xerostomia, or may aggravate it.

Keratinocytes, CD4+ and CD8+ lymphocytes, dendritics, mastocytes and macrophages play a role in pathogenesis of LP. In LP, keratinocytes are the target cells that undergo apoptosis. However, in order for the apoptosis to occur, these cells express an unknown antigen. When activated, keratinocytes secrete chemokines, which attract lymphocytes and other immune cells that aggravate LP and cause its chronicity. CD4+ lymphocytes produce interferon gamma, tumor necrosis factor-alpha (TNF-α) and interleukin 2, which are cytokines activating macrophages and CD8+ lymphocytes. Secretion of TNF-α is related to TNF-α receptor in target cells, and determines apoptosis of keratinocytes. TNF-α can enhance release of lymphocytes from blood vessels to peri-vascular areas. Moreover, it should be noted that production of TNF-α by macrophages can initiate apoptosis of basal keratinocytes and indirectly increase disintegration of basal membrane by matrix metalloproteinase 9 produced by T cells^26^. Receptors present in plasma membrane (integrins) interact with basal lamina proteins and can initiate a wide range of cell responses such as secretory processes. Thus, if membrane receptors change due to a series of environmental factors, their performance changes as well, even if other conditions are optimal (presence of ligand or proper agonist). Therefore, environmental changes such as increase in TNF-α in patients with LP can change the function of acinar cells in salivary glands. Thus, changes in signaling can change the organization of basal lamina and alter the process of Mucin 5B production in acinar mucosal cells^10^. Serous acinar cells present in the parotid gland do not produce mucin^15^. This explains the lack of a significant difference in the level of Mucin in stimulated saliva, because the main volume of stimulated saliva is secreted by the parotid glands and Mucin 5B is mainly produced by the sublingual and minor salivary glands^27^. In our study, Mucin 5B in unstimulated saliva was significantly lower in patients with OLP. The level of Mucin 5B in stimulated saliva was lower in OLP patients compared to healthy controls, but not significantly.

Changes in the level of pro-inflammatory cytokines of the saliva can change the expression of Mucin 5B by epithelial cells. Change in level of IL1, IL6, interferon gamma and TNF-α in OLP can explain change in the level of Mucin 5B in OLP patients^23^. In some patients, xerostomia has more to do with reduction in Mucin 5B and less to do with unstimulated saliva flow^10^. In OLP patients, both saliva flow and level of Mucin 5B decrease. Since Mucin 5B plays an important role in lubrication and wetting of the oral cavity, these results may explain the reason for xerostomia in OLP patients.

The level of Mucin 5B in unstimulated saliva was significantly lower in OLP patients. The level of Mucin 5B in stimulated saliva was also slightly, but not significantly, lower in OLP patients compared to healthy controls in our study. The level of Mucin 5B was higher in the serum of OLP patients. These findings may be explained as follows: an increase in the permeability of the salivary duct-blood barrier due to free oxygen radicals and other inflammatory mediators may occur in OLP patients (similar to respiratory distress syndrome), which results in an increase in serum level of Mucin 5B. On the other hand, some proteases play a role in conversion of high molecular weight mucin to low molecular weight mucin. Change in function of these proteases may occur due to inflammation in OLP and their activity may increase as such. As a result, mucin can more easily pass through the damaged salivary duct-blood barrier, compared to healthy individuals. Since this finding was seen for the first time in OLP, one or both of the above mentioned hypotheses may explain these findings. However, their confirmation requires further studies^27^.

## Conclusion

Our results show that the level of Mucin 5B in unstimulated saliva of OLP patients was significantly lower than that in healthy controls. However, the difference in level of Mucin 5B in stimulated saliva of the two groups was not significant.

Since both saliva flow and level of Mucin 5B decrease in OLP patients, and mucin plays a role in wetting and lubrication of the oral cavity, these results can, at least partly, explain the reason for xerostomia in OLP patients.

Mucin 7, like Mucin 5B, is a major mucin in saliva^28^. The current study was the first to measure the compositional change of mucin in saliva. Mucin 7 should be evaluated in future studies.
